# Adapted Immune Responses of Myeloid-Derived Cells in Fatty Liver Disease

**DOI:** 10.3389/fimmu.2018.02418

**Published:** 2018-10-18

**Authors:** Jana Hundertmark, Oliver Krenkel, Frank Tacke

**Affiliations:** Department of Medicine III, University Hospital Aachen, Aachen, Germany

**Keywords:** NASH, innate immunity, macrophages, metabolism, steatosis, fibrosis, PAMPs, lipotoxicity

## Abstract

Non-alcoholic fatty liver disease (NAFLD) is considered to be one of the most frequent chronic liver diseases worldwide and is associated with an increased risk of developing liver cirrhosis and hepatocellular carcinoma. Hepatic macrophages, mainly comprising monocyte derived macrophages and tissue resident Kupffer cells, are characterized by a high diversity and plasticity and act as key regulators during NAFLD progression, in conjunction with other infiltrating myeloid cells like neutrophils or dendritic cells. The activation and polarization of myeloid immune cells is influenced by dietary components, inflammatory signals like danger-associated molecular patterns (DAMPs) or cytokines as well as gut-derived inflammatory factors such as pathogen-associated molecular patterns (PAMPs). The functionality of myeloid leukocytes in the liver is directly linked to their inflammatory polarization, which is shaped by local and systemic inflammatory mediators such as cytokines, chemokines, PAMPs, and DAMPs. These environmental signals provoke intracellular adaptations in myeloid cells, including inflammasome and transcription factor activation, inflammatory signaling pathways, or switches in cellular metabolism. Dietary changes and obesity also promote a dysbalance in intestinal microbiota, which can facilitate intestinal permeability and bacterial translocation. The aim of this review is to highlight recent findings on the activating pathways of innate immune cells during the progression of NAFLD, dissecting local hepatic and systemic signals, dietary and metabolic factors as well as pathways of the gut-liver axis. Understanding the mechanism by which plasticity of myeloid-derived leukocytes is related to metabolic changes and NAFLD progression may provide options for new therapeutic approaches.

## Introduction

Non-alcoholic fatty liver disease (NAFLD) is a multifactorial disease affecting around 25% of the population in industrialized countries. It is commonly associated with conditions like insulin resistance, impaired glucose tolerance, dyslipidemia, hypertension, or atherosclerosis (1). The spectrum of the disease reaches from simple steatosis to non-alcoholic steatohepatitis (NASH) up to liver cirrhosis or hepatocellular carcinoma. It has been suggested that during NASH a state of chronic low-grade inflammation is induced as the consequence of multiple exogenous as well as endogenous hits originating from systemic changes as well as from liver, gut, and adipose tissue (2). One of the key components in the development of NAFLD is excess energy intake that results in an altered composition of circulating fatty acids with significantly higher levels of saturated fatty acids. Superfluous peripheral fat is stored in the form of triglycerides in adipose tissue promoting the release of adipokines such as leptin and inflammatory cytokines that regulate local as well as systemic inflammatory processes (3). Likewise, the liver as the primary metabolic organ is strongly affected by increased availability of carbohydrates and fatty acids, resulting in the accumulation and storage of toxic lipids as well as hepatic stress responses, ultimately resulting in activation of immune pathways and eventually cell death (4). Immune signals are mostly recognized through pattern recognition receptors (PRRs) by myeloid immune cells that sense pattern-associated molecular patterns (PAMPs), such as gut-derived pathogens, or danger-associated-molecular-patterns (DAMPs) released from damaged or dying cells (5). Besides cytokines, external signals including toxic lipid species or elevated levels of carbohydrates can drive activation of myeloid immune cells that may result in a broad range of immune responses. Inflammatory responses often involve chemokine-mediated recruitment of innate immune cells such as neutrophils, monocytes, or dendritic cells (DCs) to sites of inflammation, thereby affecting myeloid cell composition in metabolically important organs such as liver, gut, and adipose tissue. In response to environmental stimuli, myeloid cells are able to adapt their phenotype thus exhibiting functionally versatile roles that may contribute to initiation and progression of NAFLD. In this review, we aim to delineate the various and diverse roles myeloid cells play in the progression of fatty liver diseases and present recent insights into the crosstalk between local and systemic immune signals derived from liver, gut, and adipose tissue. The mechanisms discussed in this review have been primarily unraveled in mouse models of NAFLD and NASH, while fewer data exist from human patients supporting the relevance of these mechanisms. In addition, not all principal findings on mechanisms of macrophage activation and functional responses have been validated for macrophages in the liver, which are characterized by a remarkable heterogeneity (6). Hence, macrophages derived from bone marrow monocytes (or other tissues) and liver macrophages might not share all activation pathways. Nonetheless, the tremendous progress in recent research supports a crucial and well-orchestrated role of myeloid cells in fatty liver disease, providing exciting insights into the immune pathogenesis of NAFLD and indicating promising therapeutic targets.

## Activating signals of myeloid immune cells in fatty liver disease

### Danger signals

**Figure 1:**
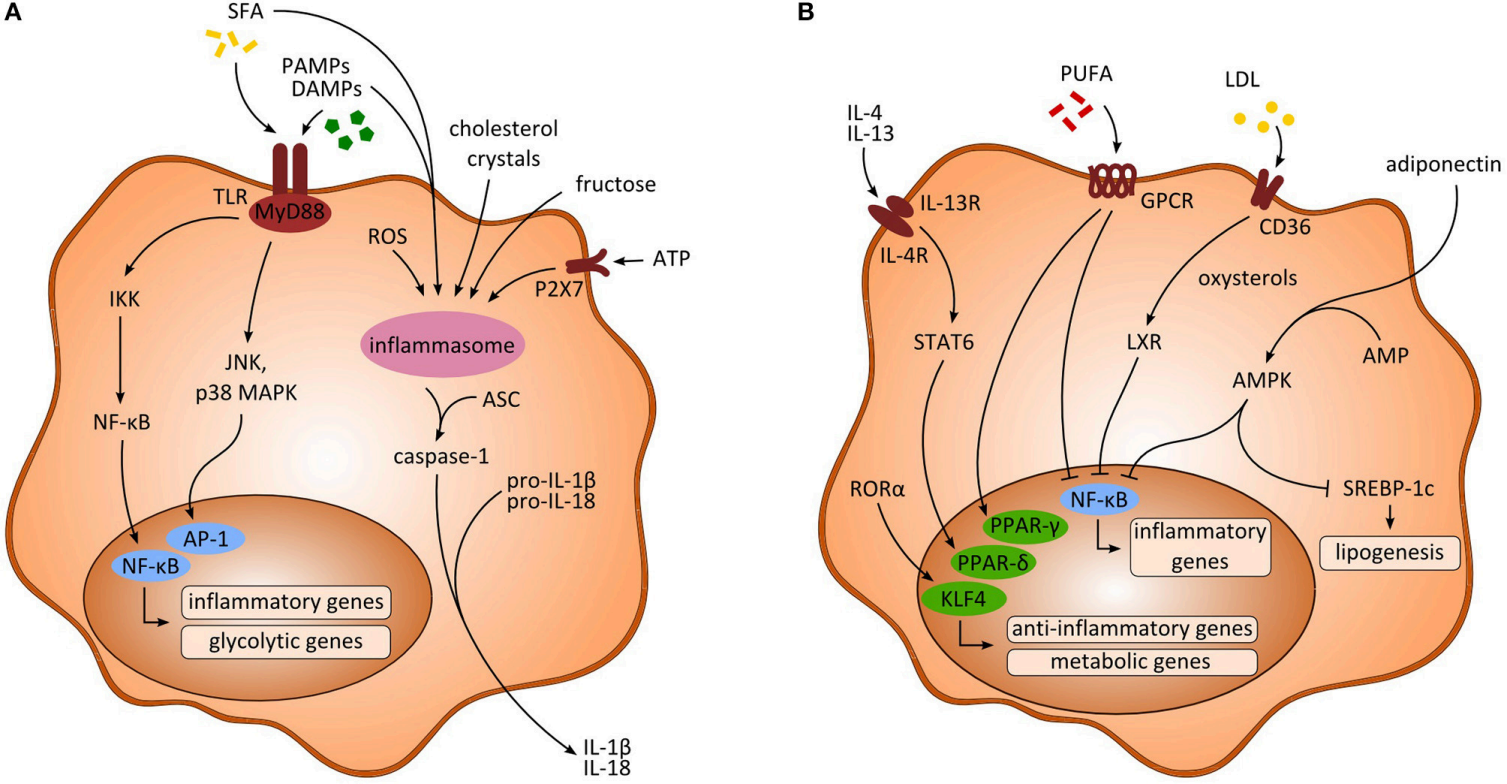
Activating signals and intracellular pathways of myeloid-derived cells. **(A)** Signaling pathways upon inflammatory activation of macrophages. Saturated fatty acids (SFA) and pathogen-associated-molecular-patterns (PAMPs) as well as danger-associated-molecular-patterns (DAMPs) activate the transcription factors NF-κB and activator protein 1 (AP-1), ultimately leading to the transcription of inflammatory and glycolytic genes. Further, activation of the inflammasome through multiple activating signals results in enhanced production of inflammatory cytokines (e.g., IL-1β, IL-18) via caspase-1. **(B)** Anti-inflammatory signaling pathways in macrophages. Anti-inflammatory cytokines (e.g., IL-4, IL-13) as well as polyunsaturated fatty acids (PUFA) and RORα lead to the transcription of PPARs and KLF4, respectively, enhancing the transcription of anti-inflammatory and metabolic genes. Moreover, inhibition of NF-κB is induced through PUFA, low density lipoprotein (LDL) and adiponectin, thereby impeding the transcription of inflammatory associated genes. AMP, adenosine monophosphate; AMPK, AMP-activated protein kinase; ASC, apoptosis-associated speck like protein containing a caspase recruitment domain; ATP, adenosine triphosphate; IKK, inhibitory κB kinase; LXR, liver X receptor; MAPK, mitogen-activated protein kinase; ROS, reactive oxygen species.

During NAFLD development, endogenous DAMPs that are released from damaged or stressed cells initiate sterile inflammation and contribute to the activation of myeloid cells (7, 8). The pathology of NASH is linked to a chronic state of sterile inflammation induced by high levels of DAMPs that lead to the activation of PRRs (Figure 1A). Molecules that have been recognized as danger signals in fatty liver disease comprise nuclear factors e.g., high mobility group box protein (HMGB1), nucleic acids, lipid mediators, histones, ATP, and uric acid (9). Additionally, gut-derived PAMPs such as lipopolysaccharide (LPS), bacterial DNA, or peptidoglycans can enter the enterohepatic circulation due to increased intestinal permeability and hence stimulate immune cells in the liver (5). Inflammasome-dependent mechanisms further control diversity and composition of the gut microbiota, and NAFLD-associated dysbiosis facilitates DAMPs and PAMPs entering portal circulation, thereby promoting inflammatory responses in the liver and aggravating steatohepatitis (10).

DAMPs are typically recognized by toll-like receptors (TLRs) or P2X purinoceptor 7 (P2X7) that in turn result in activation of the inflammasome (9). In mouse models of NASH, an increase in accumulation of HMGB1, uric acid, ATP, and exogenous LPS has been discovered along with increasing populations of pro-inflammatory macrophages (11). Consistent with this, increased extracellular ATP released by damaged cells was shown to stimulate the inflammasome via P2X7 receptor signaling in NASH models. Interestingly, ATP released from hepatic cells upon liver injury further resulted in the recruitment of peritoneal cavity macrophages exhibiting an anti-inflammatory like phenotype (12). HMGB1 and IL-33 are released from dying hepatocytes and showed significant upregulation in mice during NASH progression (13), in which HMGB1 activates TLR4 and the adaptor protein myeloid differentiation primary response 88 (MyD88) (14). HMGB1 was further shown to be necessary for neutrophil recruitment in acute liver injury (15). Similarly, elevated levels of HMGB1 were found in obese adipose tissue (16) and may aggravate inflammatory activation of adipose tissue macrophages. Moreover, in response to cytokine stimulation through LPS, TNF or TGF-β, HMGB1 was shown to be released by activated macrophages or DCs that contributed to the activation of pro-inflammatory pathways (17). Further, the extracellular alarmins S100A8 and S100A9 were shown to promote inflammation in monocytes and macrophages *in vivo* as well as *in vitro* via the receptor advanced glycation end-products (RAGE) and TLR4 dependent pathways in mice and men (18, 19). Most receptors targeted by DAMPs can also be activated by PAMPs, which is of special interest in the context of alterations in the gut microbiome and bacterial defense mechanisms during NAFLD. Intestinal permeability in NASH further favors translocation of microbial products such as bacterial DNA, LPS from gram-negative bacteria or β-Glucan from fungi that may act as microbial-associated molecular patterns (MAMPs) or PAMPs. Indeed, several studies indicate a high relevance of elevated gut-derived LPS and TLR signaling in mouse models of NASH (20–22). Additionally, altered bile acid homeostasis might promote the progression of NAFLD by shaping macrophage function (23). Bile acids are important mediators of lipid and glucose metabolism in the liver acting through nuclear receptors, farnesoid X receptor (FXR), and G-protein-coupled-receptor TGR5. In Kupffer cells (KCs), sensing of bile acids through TGR5 resulted in secretion of anti-inflammatory cytokines. Moreover, activation of TGR5 was also associated with inhibition of the inflammasome (24).

### Lipotoxicity

Bioactive lipids have substantial functions in energy homeostasis, cell communication, regulation of inflammation, and maintaining structural integrity. In NAFLD an increased availability of dietary lipids, increased hepatic *de novo* lipogenesis and increased lipolysis in adipose tissue jointly lead to accumulation of triglycerides in hepatocytes. A dysbalance of extra- and intracellular lipid composition caused by excess free fatty acids may result in the accumulation of toxic lipids with ensuing cellular injury involving organelle dysfunction or cell death (25). Toxic lipids are known to affect the endoplasmic reticulum (ER) by causing ER stress and activation of the unfolded protein response, whereas in mitochondria, excess lipids contribute to the production of reactive oxygen species (ROS) (25). Circulating fatty acids in the blood stream mainly comprise saturated fatty acids such as palmitic or stearic acid that are thought to induce inflammation via TLR dependent pathways, mainly involving TLR4. *In vitro*, palmitate induced TLR2 and TLR4 dependent pro-inflammatory signaling in both monocytes and macrophages (26). However, recent findings indicated that palmitate does not act as a TLR4 agonist directly, but that TLR4-dependent priming is necessary for immune signaling through palmitate (27). A recent study further investigated the effect of palmitate induced death receptor 5 signaling on the secretion of extracellular vesicles from hepatocytes and showed that these vesicles promoted a pro-inflammatory phenotype in macrophages (28). Further, pro-inflammatory hepatic macrophages release ROS upon stimulation with palmitate through internalization of TLR4/MD2 complexes (29). However, short chain fatty acids (SCFA) protect against diet-induced obesity by inducing a PPAR-γ mediated switch to fat oxidation (30). Additionally, mouse models showed elevated levels of ceramides enhanced by the cytokines IL-6 and IL-10. Consistently, inhibition of ceramide synthesis reduced steatosis in hepatocytes (31). In the liver, free fatty acids have been shown to activate KC through TLR2 and TLR4 signaling (32). Further, intracellular storage of lipids has been observed in KCs during experimental NASH that was associated with an inflammatory phenotype *in vivo* as well as *in vitro* (33, 34).

In contrast, polyunsaturated fatty acids (PUFA) such as omega-3 and omega-6 fatty acids appear to play a protective role regarding inflammation (Figure 1B). In macrophages, docosahexaenoic acid has been shown to activate PPAR-γ and AMP-activated protein kinase (AMPK) thus inhibiting expression of the transcription factor nuclear factor kappa beta (NF-κB) (35, 36). Docosahexaenoic acid and eicosapentaenoic acid bind to G-protein-coupled-receptor 120 (GPR120) on macrophages and in turn inhibit NF-κB and improve insulin resistance (37). Interestingly, macrophages and other myeloid cells (monocytes, neutrophils) also express receptors for medium chain fatty acids, especially GPR84, which is induced by LPS, high glucose concentrations and oxidized LDL (38). The functional consequences of this pathway are not fully understood. Potentially, medium chain fatty acids can amplify inflammatory and/or fibrogenic responses via GPR84 in macrophages (38, 39).

Moreover, lipotoxicity essentially involves mitochondrial dysfunction due to elevated levels of free fatty acids leading to increased production of ROS as well as lipid peroxidation. In the mitochondria, redox nodes (e.g., NADPH/NADP+) contribute to oxidative phosphorylation and also balance the redox state of the cell. In this context, it was shown that mice deficient in NADPH were protected from dysfunction in oxidative phosphorylation and NASH induced by a high-fat diet (40). Increased levels of ROS in NAFLD result in depletion and inhibition of antioxidative molecules and enzymes such as thioredoxin and glutathione. Accordingly, experimental evidence suggests that treatment with antioxidants, e.g., glutathione, may prevent the progression toward NASH by reducing hepatic oxidative stress (41).

### Glucotoxicity

Aside lipids, also an increased uptake of sugars and other carbohydrates is associated with sustained NAFLD progression (1). Most interestingly, a few studies have established a link between sugars and activation of myeloid immune cells. Fructose has been shown to induce NOD-like receptor protein 3 (NLRP3) inflammasome formation in human macrophage cell lines via thioredoxin-interacting protein (TXNIP) to mitochondria shuttling, resulting in an increased production of ROS as well as IL-1β and IL-18 (42). Moreover, upregulation of various glucose transporters (GLUT) has been described during monocyte to macrophage differentiation (43). In line, increased GLUT1 expression leads to an upregulated pentose-phosphate pathway and an inflammatory macrophage phenotype, characterized by a high expression of CCL2, CXCL2, TNF-α, and IL-6 (44). GLUT3 further mediates glucose uptake following hypoxia in human macrophages, leading to *de novo* lipogenesis and subsequent lipid droplet accumulation (45).

### Inflammatory cytokines

One key mechanism of relaying inflammatory processes between fat, liver, and gut during NAFLD is the release of cytokines such as IL-1β, IL-6, IFN-γ, and TNF-α, which aggravate local inflammation, thereby worsening insulin resistance and triglyceride accumulation in fat and liver tissue. Inflammatory cytokines and chemokines, particularly TNF-α, IL-8 and CCL3, were also shown to be correlated with disease severity in human NAFLD patients (46), and myeloid immune cells, such as monocytes, neutrophils, and DC are well-known sources of both cytokines and chemokines (47).

IL-1β is released by myeloid immune cells following inflammatory polarization and inflammasome activation (48). In the liver, KCs are well-known sources of IL-1β and IL-18 during NAFLD (49, 50), and it has been shown that cholesterol treatment induces IL-1β and IL-18 production in both KCs and monocyte derived macrophages in a dose dependent manner (51). In line, also adipose tissue macrophages and neutrophils secrete IL-1β, thereby promoting inflammation during NAFLD progression (52). IL-6 is known to be secreted by macrophages and DC during NAFLD (53, 54), and has been associated with increased risk for insulin resistance in men (55). TNF-α is another often named inflammatory cytokine in NAFLD (56), and can be secreted by neutrophils (57), monocytes, adipose tissue macrophages (58), KCs (59), and DCs (53). Although TNF-α secretion enhances local inflammation thereby worsening insulin resistance and hepatic steatosis (60), early studies in humans exploring TNF-α blockade as a therapeutic target in metabolic diseases did not show beneficial effects (61). Another important group of cytokines are interferons, which have been shown to be relevant during NAFLD progression (62). While interferon secretion is typically associated with T, NK, and NKT cells, it induces an inflammatory phenotype in macrophages (13). Moreover, cytosine-phosphate-guanosine (CpG) microbial motif stimulation mediates autocrine type I IFN signaling, and subsequently induces fatty acid oxidation in plasmacytoid DCs (63). Furthermore, Galectin-3, a lectin secreted by macrophages is increased in obesity and stimulates pro-inflammatory pathways in adipose tissue (64).

## Intracellular responses of myeloid immune cells upon activation

### Intracellular signaling

Intracellular pathways following activation by PAMPs and DAMPs mainly involve the activation of PRRs, e.g., TLRs, that immediately lead to an inflammatory response and, accordingly, several studies demonstrated TLR upregulations associated with metabolic diseases. Upon binding to its corresponding ligand TLRs associate with MyD88, which results in an activation of c-Jun N-terminal kinase (JNK), inhibitor of nuclear factor kappa-B kinase 2 (IKK2) and mitogen-activated protein kinase (MAPK) p38 (Figure 1A). As a consequence, expression of NF-κB and activator protein 1 (AP-1) is upregulated and ultimately results in transcription of pro-inflammatory cytokines such as IL-6 or TNF-α (5, 65) (Figure 1A). Consequently, interfering with NF-κB signaling by pharmacological inhibition of IKK2 significantly reduced hepatic steatosis and inflammatory responses in NASH mice (66). Diet-induced activation of JNK was further shown to be enhanced by mixed-lineage kinase 3 (MLK3) thus increasing hepatic steatosis as well as insulin resistance (67). In line, expression of JNK in adipose tissue macrophages was found to be necessary for the induction of insulin resistance and inflammation in obese mice (68). Danger signal HMGB1 further enhances LPS induced pro-inflammatory activation of macrophages through phosphorylation of MAPK p38 and activation of NF-κB (69).

In addition, hematopoietic knockout of TLR4 in obese mice demonstrated a crucial role of TLR4 in inducing insulin resistance in liver and adipose tissue, especially in KCs and macrophages (70). In murine KCs, TLR4 further promotes ROS dependent activation of X-box binding protein thus regulating diet-induced NASH (71). Moreover, translocated bacterial DNA binds to TLR9 expressed by KCs thereby enhancing secretion of IL-1β, which subsequently induces steatosis in mice and promotes fibrogenesis (50).

### Activation of the inflammasome

Recent studies suggest an important role of the inflammasome in the progression of NAFLD, as it is expressed in innate immune cells including monocytes, macrophages, DCs, and neutrophils. Inflammasomes are intracellular (cytosolic) multiprotein oligomers that recognize danger signals via the nucleotide oligomerization domain (NOD)-like receptor (NLR). The sensing of PAMPs and DAMPs results in complex formation of the NLR sensor, ASC and pro-caspase-1. After activation, caspase-1 leads to maturation and subsequent secretion of inflammatory IL-1β and IL-18 (72) (Figure 1A). In experimental NASH, mice fed a high-fat diet showed accumulation of cholesterol crystals in KCs leading to activation of the inflammasome (73). Similarly, *in vitro* exposure of phagocytes to cholesterol crystals induced assembly of NLRP3 inflammasomes (74). In contrast, lack of the NLRP3 inflammasome results in decreased levels of IL-18 and other cytokines in the intestine and is related to an altered gut microbiota and derangement of the gut-liver-axis (10, 75). Consistently, NLRP3 blockade improved NAFLD in obese mice presumably by inhibiting inflammasome activation mediated by cholesterol crystals in myeloid cells (51). Of note, a derivate of cholesterol, 27-hydroxycholesterol, was shown to reduce cholesterol accumulation in KCs and thereby attenuated steatosis in mice (76). Interestingly, the NLRP3 inflammasome also seems to be critical in Western diet mediated reprogramming of myeloid immune cells, which suggests a possible role in inducing trained immunity in these cells (77). Wen et al. further demonstrated that the saturated fatty acid palmitate induced NLRP3-ASC inflammasome assembly in macrophages involving AMPK and ROS signaling (78). Aside palmitate, also stearate could induce assembly of the inflammasome in human macrophages, whereas unsaturated fatty acids as oleate and linoleate prevented activation of NLRP3 (79). Inflammasome complexes are also expressed in human KCs and respond to common liver pathogens as *S. thyphimurium* and *F. novicida*, whereas hepatitis B virus (HBV) infection inhibited inflammasome formation (80). Deficiency of caspase-1 and caspase-11 in macrophages reduces hepatic inflammation through reduced formation of cholesterol crystal and enhanced cholesterol efflux in obese mice (81).

### Cellular metabolism

Adaptations in the cellular metabolism of myeloid immune cells essentially shape their plasticity and functionality through the interaction of several transcription factors, signaling molecules, posttranscriptional, and epigenetic regulation. Varying availability of nutrients requires metabolic reprogramming in myeloid cells to ensure appropriate activation and function of metabolic processes (e.g., glycolysis, citric acid cycle).

Inflammatory activation of macrophages (e.g., through LPS or IFN-γ) is associated with enhanced aerobic glycolysis leading to increased lactate production as well as activation of the pentose phosphate pathway (82). The transcription factors hypoxia-inducible factor-1α (HIF-1α) and HIF-2α control the transcription of inflammatory associated NOS2 and anti-inflammatory arginase-1 (ARG1), respectively. Increased expression of inducible nitric oxide synthase (iNOS) in inflammatory macrophages and DCs is linked to enhanced production of nitrogen species that may inhibit mitochondrial respiration. The increased influx of glucose in pro-inflammatory macrophages is regulated by HIF-1α, whereas loss of HIF-1α is associated with reduced secretion of inflammatory cytokines (83). This suggests a possible role of HIF-1α in the pathology of NAFLD, as chronic hypoxia was shown to directly trigger NAFLD by increasing macrophage accumulation in adipose tissue (84). In DCs, LPS stimulation drives glucose oxidation leading to enhanced synthesis of phospholipids and production of pro-inflammatory cytokines (85). On the other side, during homeostasis resting cells show increased consumption of glucose as well as fatty acids that trigger activation of citric acid cycle and oxidative phosphorylation (82). Furthermore, the carbohydrate kinase like (CARKL) protein controls metabolic reprogramming via the pentose phosphate pathways and was found to be downregulated in anti-inflammatory macrophages in both mice and humans (86). Increased glucose metabolism in anti-inflammatory macrophages is promoted through mTORC2 and IRF4 signaling that is essential for metabolic remodeling (87).

Moreover, regulation of lipid metabolism as well as transcription of inflammatory and anti-inflammatory genes significantly shapes the functional phenotype of myeloid cells. Inflammatory signaling in NAFLD is associated with activation of NF-κB by saturated fatty acids that activate sterol regulatory element-binding protein-1 (SREBP-1), which then leads to increased lipogenesis via acetyl-CoA carboxylase (88, 89). Polarization toward an anti-inflammatory phenotype can be regulated by different ways. The scavenger receptor CD36 takes up oxidized low density lipoprotein (LDL), which is then converted to oxysterol by cytochrome P450 oxidase. Next, the nuclear liver X receptor (LXR) is activated by oxysterols and in turn leads to inhibition of NF-κB signaling and hence an attenuation of inflammation (90) (Figure 1B). Additionally, levels of adenosine monophosphate (AMP) can be elevated due to metabolic factors (e.g., adiponectin) and cause activation of AMP kinase (AMPK) that inhibits Acetyl-CoA carboxylase. This results in decreased levels of Malonyl-CoA and consequently increased fatty acid oxidation and inhibition of SREBP-1C (Figure 1B). Activation of AMPK was shown to induce an anti-inflammatory phenotype in macrophages through inhibiting inflammatory polarization and crucially contributes to immune function in macrophages (91).

Nuclear factors peroxisome proliferator-activator receptors (PPARs) are activated by fatty acids or their intermediates and regulate transcription of metabolic processes. In mice, PPAR-γ specific knockout in macrophages protected against diet-induced hepatic steatosis (92). In addition, retinoic acid receptor-related orphan receptor-α (RORα) regulates hepatic lipid homeostasis by reducing PPAR-γ activity. In the liver, it was recently shown that RORα increases anti-inflammatory polarization of liver macrophages through Kruppel-like factor 4 (KLF4) and accordingly ablation of RORα in myeloid cells predisposed mice to high-fat diet-induced NASH (93). Anti-inflammatory cytokines (e.g., IL-4, IL-13) released by adipocytes or hepatocytes activate PPAR-δ expression via STAT6 signaling and thereby induce an anti-inflammatory phenotype in adipose tissue as well as hepatic macrophages (94) (Figure 1B). In line, myeloid specific deletion of PPAR-δ resulted in increased insulin resistance and development of steatohepatitis (95).

However, chronic low grade inflammation in NAFLD is perpetuated by several factors (e.g., dietary fats, chronic changes to the gut microbiome, persisting changes in metabolism, adipose tissue mediators), which likely explain why anti-inflammatory pathways fail to achieve adequate resolution of NAFLD (unlike after an acute injury to the liver). In this context, the dual inflammatory and pro-resolving role of Kupffer cells and infiltrating macrophages is of great importance (96). Reprogramming of inflammatory macrophages toward an anti-inflammatory phenotype might represent an interesting therapeutic target to strengthen anti-inflammatory signaling and improve NAFLD progression, yet it is unclear if this would be able to overcome the inflammatory signals, if the injurious trigger (e.g., overnutrition) persists.

Cellular metabolism has further been shown to be controlled by epigenetic factors that are involved in the development and progression of NAFLD (97). In obese subjects, progression toward NASH was linked to modified methylation of genes associated with insulin metabolism (98). Moreover, epigenetic alteration of hepatic DNA were shown to significantly affect energy metabolism in mitochondria and influence fibrogenic signaling with increasing age (99).

## Myeloid cell composition, differentiation, and polarization in different compartments

### Liver

**Figure 2:**
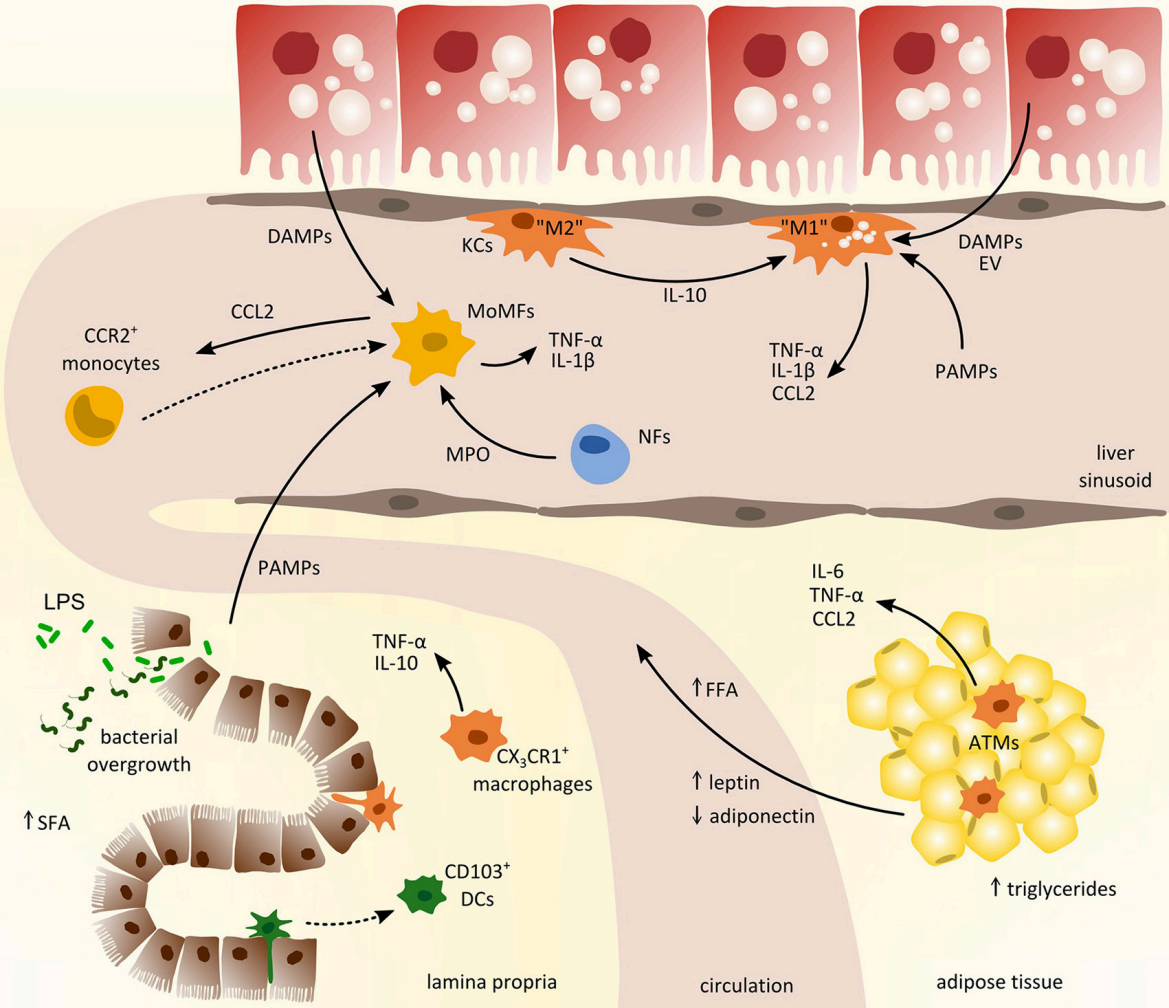
Cross-talk of myeloid-derived cells from different compartments in the pathogenesis of non-alcoholic fatty liver disease (NAFLD). Inflammatory activation promoting the progression of NAFLD involves the interaction between liver, gut, and adipose tissue. From the gastrointestinal tract (left lower corner), pathogen-associated-molecular-patterns (PAMPs) enter the circulation due to increased intestinal permeability and promote hepatic inflammation together with local danger-associated-molecular-patterns (DAMPs). In adipose tissue (right lower corner), chronic inflammation induces the release of pro-inflammatory cytokines (e.g., IL-6, TNF-α) and free fatty acids (FFA) that trigger hepatic injury and activation of hepatic macrophages such as monocyte-derived macrophages (MoMFs) and tissue-resident Kupffer cells (KCs). Activated macrophages in the liver produce CCL2 (and other pro-inflammatory cytokines) to recruit circulating monocytes from the blood that contribute to sustained inflammation. ATMs, adipose tissue macrophages; DCs, dendritic cells; EV, extracellular vesicles; LPS, lipopolysaccharide; MPO, myeloperoxidases; NFs, neutrophils; SFA, saturated fatty acids.

The liver harbors multiple distinct subsets of macrophages and DCs. Two major types of macrophages are located in the liver: monocyte-derived macrophages and yolk sac-derived tissue-resident KCs. Although myeloid cells in general and macrophages in particular exhibit a remarkable heterogeneity in the liver (6), many studies oftentimes simplistically denote these cells as KCs. The unique vascular supply of the liver and the sinusoidal localization of many hepatic myeloid cells imply that these cells are among the first to encounter gut-derived PAMPs via the hepatic port vein, but are also exposed to local DAMPs (Figure 2). During the progression of NAFLD, myeloid cell composition changes fundamentally. In the early phase of NAFLD associated inflammation, the most important immune cell population in the liver are the tissue resident KCs (100), which survey their surrounding for stress or danger signals like e.g., DAMPs released by steatotic hepatocytes. Depletion of KCs was consequently shown to ameliorate NASH in mice, underlining their importance in the progression of the disease (60). Interestingly, during NASH, a preponderance of anti-inflammatory polarized KCs was found that initiated apoptosis of inflammatory KCs by the secretion of IL-10 (101) (Figure 2). Moreover, mice supplied with probiotics, e.g., *L. paracasei*, exhibited enhanced anti-inflammatory macrophages in the liver that were involved in attenuation of hepatic steatosis in NASH (21). These results highlight that the state of polarization of KCs and other macrophage populations is crucial in the progression of NAFLD. Following DAMP stimulation release of TNF-α by hepatic macrophages is necessary to activate NF-kB signaling in hepatocytes and CXCL1 production for the recruitment of neutrophils (102). In mice with steatohepatitis, diet-derived lipids activated pro-inflammatory KCs that in turn lead to reduced hepatic NKT cells due to cell death (103).

Upon inflammation monocytes are primarily recruited via CCR2-CCL2 as well as CXCR3 and CXCL10 dependent signaling (6, 104). Accordingly, the infiltration of Ly-6C+ monocytes has been identified as a crucial factor in the progression toward NASH and fibrosis in mice (100, 105). The most widely explored pathway of recruiting inflammatory and fibrogenic monocytes to injured liver is via the chemokine CCL2 (106), which can also be targeted therapeutically in experimental models of steatohepatitis (107). Besides CCL2 secreted by KCs and steatotic hepatocytes, TNF related apoptosis inducing ligand (TRAIL) might be involved in the recruitment of circulating monocytes (108). Additionally, damaged hepatocytes may release extracellular vesicles containing CXCL10 or ceramides, thereby initiating recruitment of myeloid cells to the liver (109, 110). Ceramides were shown to affect macrophage chemotaxis via sphingosine-1-phosphate; consequently, inhibition of sphingosine-1-phosphate attenuated steatosis in experimental NASH (111). Moreover, dysregulation of lipid synthesis due to steatosis can also lead to accumulation of toxic lipids in KCs that in turn exhibit a pro-inflammatory phenotype, as characterized by the secretion of inflammatory cytokines and chemokines as well as enhanced recruitment of lymphocytes (33). Interestingly, arginase 2 deficient mice, lacking anti-inflammatory macrophages, develop spontaneous steatosis and show characteristics of early steatohepatitis without high-fat diet feeding (112). Furthermore, CD44 appears to be involved in the induction of a pro-inflammatory phenotype in hepatic macrophages thereby aggravating progression toward NASH in mice and men (113). Additionally, palmitate-induced release of extracellular vesicles (containing TRAIL) from hepatocytes was shown to promote a pro-inflammatory phenotype in macrophages, thus exposing an interesting therapeutic target for NASH in patients (28).

Neutrophils are mainly recruited toward areas of inflammation via the CXCR1/2 receptors and CXCL1/2 axis (in mice) or IL-8 (in humans), respectively (47). The induction of CXCL2 secretion by hepatic cells has been shown to depend on both TLR2 and S100A9 (114). Furthermore, myeloperoxidases secreted by neutrophils are increased in NASH patients (115). Myeloperoxidases were shown to be toxic to macrophages, thereby contributing to the progression of inflammation and insulin resistance (57, 116). Consequently, in NAFLD models mice lacking myeloperoxidases showed reduced hepatic inflammation as well as improved insulin resistance (115). In addition, neutrophils may contribute to suppression of bacterial infections via extracellular traps activated by the release of DAMPs in liver injury (117, 118).

Recently, an influx of inflammatory DCs has been demonstrated in experimental NASH that was associated with a regulatory phenotype contributing to clearance of apoptotic cells and thus limiting inflammation (53). Furthermore, inflammatory DCs expressing CX_3_CR1 were shown to contribute to sustained inflammation in mice with diet-induced NASH and were linked to increased levels of TNF-α (119). Moreover, capability of hepatic DCs to respond to immunogenic stimuli seems to be dependent of their lipid content and concentration in mice and men (120).

### Adipose tissue

During fatty liver disease progression, the adipose tissue is not simply an organ for excess lipid storage. In fact, adipose tissue actively contributes to systemic metabolic disturbances and to inflammatory changes in NAFLD, not only in the adipose tissue itself but also in the liver. Compared to homeostatic conditions the composition as well as the number of myeloid immune cells in adipose tissue is altered in obese patients. Increased expression of CCL2 in adipocytes leads to monocyte recruitment and accumulation of macrophages in adipose tissue (3). Macrophage infiltration then results in enhanced accumulation of triglycerides in adipocytes further enhancing adipose tissue inflammation. The infiltration of monocytes is typically accompanied by an increase in adipose DCs and an early influx of neutrophils. In addition to an increased infiltration of macrophages in adipose tissue under obese conditions, an altered inflammatory phenotype can be observed in these cells characterized by enhanced TNF production (Figure 2). In this context Chung et al. showed that induction of inflammation is dependent on the interaction of adipocytes and macrophages through α_4_-integrin (58). It was further demonstrated that gut-derived LPS induced inflammatory pathways in adipose tissue through TLR signaling and contributed to the recruitment of macrophages by increasing CCL2 expression in adipocytes (22). In line with this, ablation of TLR4 in adipose tissue reduced the infiltration and macrophages that exhibited an M2 like phenotype (121).

Adipose tissue inflammation is further driven by an infiltration of neutrophils. Neutrophils are characterized by high numbers of cytoplasmic granules containing neutrophil elastase, myeloperoxidase, lysozymes, and other antibacterial defensins. Following tissue infiltration, neutrophils also secrete cytokines as IL-1β, IL-8, and TNF-α, inducing local inflammatory processes, especially during early stages of adipose tissue inflammation following high-fat diet feeding (52, 57).

In experimental NASH, adipose tissue derived leptin has been shown to promote KC activation in the liver via iNOS and NADPH oxidase that cause oxidative stress and was further linked to increased levels of TGF-β (122, 123). High levels of leptin also enhanced expression of the LPS receptor CD14 via STAT3 signaling in KCs, thus increasing their reactivity to low doses of gut derived bacterial LPS and ultimately leading to increased hepatic inflammation (124). On the other hand, adiponectin which is reduced in NAFLD, had anti-inflammatory effects on macrophages and induced reduction of TNF-α (125). Furthermore, insulin like growth factor 1 (IGF1) is produced by IL-4 mediated alternatively activated macrophages, and IGF1 receptor (IGF1R) positive macrophages attenuate adipose tissue inflammation (126). In obese mice, exosomes containing miRNA (e.g., mi-R155) secreted by adipose tissue macrophages were shown to promote glucose tolerance and insulin resistance suggesting a possible role in in the progression of NAFLD (127), especially regarding crosstalk between organs (128). Moreover during obesity, adipose tissue derived S100A8 and S100A9 stimulated IL-1β production in adipose tissue macrophages through TLR4 and NLRP3 dependent pathways then promoting an increase of myeloid precursors in the bone marrow via IL-1R in mice (129).

### Gut

Evidence from mice and men demonstrate the great importance of the cross-talk between liver and the intestine in the development of metabolic diseases (130, 131). NAFLD is often accompanied by an altered gut microbiota and bacterial overgrowth that is strongly associated with increased intestinal permeability as well as enhanced pathological bacterial translocation. In the gut, mononuclear phagocytes, comprising macrophages, DCs, and neutrophils, are tightly controlled to ensure intestinal homeostasis (132). During homeostasis and inflammation, intestinal macrophages, characterized by their expression of F4/80 and CX_3_CR1, secrete IL-10 and low levels of TNF-α stimulating regulatory T-cells as well as newly infiltrating monocytes (133) (Figure 2). Myeloid cells were further shown to contribute to the progression of bacterial infections during liver fibrosis by production of IFN-γ and increased secretion of IL-10 (20). Studies in CX_3_CR1-deficient mice further depict a contribution to intestinal homeostasis through intestinal macrophages thus ameliorating diet-induced steatohepatitis (134). Moreover, intestinal macrophages exert a high phagocytic activity and may be also involved in limiting bacterial translocation (135). Intestinal CD103^+^ DCs are mainly responsible to transport bacteria to mesenteric lymph nodes. In line, in CCl_4_ induced liver cirrhosis in rats enhanced bacterial translocation was linked to increased levels of CD103^+^ DCs (136). DCs in the gut are further known to express tight junction proteins (e.g., occludin) and thereby influence bacterial translocation in response to oxidative stress and inflammatory stimulation (137).

Several studies indicate a high relevance of gut microbiota dysbiosis and imbalanced microbiota-produced metabolites (e.g., SCFA or ethanol) through increasing the susceptibility for inflammatory liver diseases (138). This is underlined by a recent study in which germ free mice inoculated with feces from patients suffering from NAFLD developed more severe NASH after a high-fat diet compared to control mice (139). Further, gut-derived bacterial metabolites (e.g., tryptamine and indole-3-acetate), that are depleted upon high-fat feeding can mediate inflammation in macrophages and hepatocytes by lowering levels of pro-inflammatory cytokines as TNF-α, IL-1β, and CCL2 (140). Studies by Caesar et al. could also demonstrate that gut microbiota derived LPS promotes macrophage recruitment into adipose tissue and pro-inflammatory TNF-α expression (141). Early evidence by Turnbaugh et al. demonstrated that the gut microbiota of obese mice further possesses an increased ability to harvest energy from their diet underlining its major role in metabolism and hence metabolic diseases (142). Moreover, in recent metagenomic studies, hepatic steatosis was associated with a reduction in microbial gene richness with an increase in *Proteobacteria* and elevated levels of the microbial metabolite phenylacetic acid that promotes hepatic lipid accumulation (143). Even though several microbial metabolites have been shown to strongly influence the progression of NAFLD, the diverse interactions between host and microbiome most likely comprise more complex factors and require further analyses.

Moreover, SCFA are significantly involved in the pathology of NAFLD, as they are the major fermentation products of the gut microbiome including acetate, propionate, and butyrate. Compared to long chain fatty acids that lead to the initiation of inflammatory pathways, short chain fatty acids seem to have a countervailing effect on the progression of NAFLD. Diet-derived SCFA inhibit the expression of PPAR-γ and hence lead to the activation of AMPK reducing glycolysis and lipogenesis (30). These effects are primarily mediated via the GPR43 receptor. However, the precise role of the potential anti-inflammatory effects SCFA in fatty liver disease and their potential therapeutic value need to be investigated more detailed.

## Modulating myeloid-derived cells in fatty liver disease

### Therapeutic modulation of activating signals

The thorough understanding of activating pathways and functional roles of myeloid cells in fatty liver diseases offers the opportunity to develop novel treatment strategies. Therapeutic targeting of activating signals of myeloid cells, especially macrophages, has been a promising approach to treat liver diseases. Recent trials target bile acid receptors to influence the immune phenotype of macrophages. In mice, a dual TGR5/FXR agonist, INT-767, was shown to protect against steatohepatitis and increase numbers of anti-inflammatory monocytes and macrophages (144). Further, the semi-synthetic bile acid analog obeticholic acid is a strong FXR agonist with promising results from an early phase clinical trial in patients with NASH (145). While FXR antagonism has many beneficial effects on metabolism, it was also shown to reduce activation of murine KCs *in vitro* (146). The multi-ligand PRR RAGE (receptor for S100 proteins and HMGB1) is also considered as potential therapeutic target, as it is implicated in several chronic inflammatory diseases. However, blocking RAGE in rats could not inhibit LPS induced hepatic oxidative damage (147). Additionally, dietary lipid composition can be modulated to counteract inflammatory processes. Although NASH patients show decreased levels of n-3 PUFA, clinical trials supplementing PUFAs were not able to prove improvement of steatosis or fibrosis (148). Further there have been investigations interfering with TNF-α related signaling to treat alcoholic hepatitis or NASH by using monoclonal antibodies or inhibiting TNF-α (e.g., pentoxifylline or etanercept), however such drastic interventions are unlikely to become clinical routine for long-term treatment due to safety concerns (149, 150).

Interfering with gut-derived signals, particularly PAMPs, might also reduce activation of myeloid cells in fatty liver disease in the liver as well as in the gut. Microbiome-based strategies to treat NAFLD predominantly include the use of antibiotics, prebiotics or probiotics. Studies by Jiang et al. demonstrate that combined treatment with bacitracin, neomycin, and streptomycin interferes with the gut microbiota and regulates NAFLD progression through decreased FXR signaling as well as reduced ceramide levels (151). Another novel approach involves fecal microbial transplantation to modulate the gut microbiota. In a recent study, fecal microbial transplantation was shown to limit high-fat diet-induced NAFLD (152). Similarly, intestinal microbiota transfer determined the susceptibility to NASH induced by high-fat diet feeding in mice (153). In addition, orlistat, a gut lipase inhibitor, can attenuate diet-induced obesity by limiting absorption of dietary fatty acids from the gut (154).

### Therapeutic modulation of intracellular responses in myeloid cells

Key intracellular signaling pathways in NASH are intensively investigated at the moment and, amongst others, include PPARs as they essentially regulate glucose and lipid metabolism. Agonists for PPAR-γ and PPAR-δ (e.g., elafibranor) reduced inflammation and fibrosis in mouse models of NAFLD (155) and also demonstrated promising results in an early clinical trial (156). These findings were associated with reduced release of pro-inflammatory cytokines such as TNF-α (94, 156). Further, a pan-PPAR agonist, IVA337, was shown to markedly improve NASH features in mice (157). PPAR-γ agonists further ameliorated lipid-induced polarization of inflammatory KCs in mice resulting in an improvement of steatohepatitis (158) and also reduced components of NASH in a long term clinical study (159). Many other drugs affecting intracellular inflammatory or metabolic pathways are currently being evaluated as potential treatments for NAFLD (4). Among them, the apoptosis signal-regulating kinase 1 (ASK1) inhibitor selonsertib blocks inflammatory signaling (JNK and p38 MAPK) in KCs (160), which might explain antifibrotic signals observed in an early clinical trial (161).

### Therapeutic modulation of myeloid cell composition, differentiation, and polarization

The interplay between recruited and tissue-resident myeloid-derived cells allows the interference with recruiting signals (e.g., chemokines) to reduce myeloid cell accumulation. Inhibition of hepatic macrophage infiltration can be realized by specifically blocking distinct chemokine receptors, e.g., through cenicriviroc, a dual antagonist against the chemokine receptors CCR2 and CCR5 (162–164). Due to the primarily inflammatory and fibrogenic phenotype of infiltrating monocyte-derived macrophages in steatohepatitis (165), this principle was found effective in reducing fibrosis in animal models (166) as well as in NASH patients treated for 1 year with cenicriviroc (167). However, such a strategy would not directly affect KCs or non-CCR2 dependent myeloid cells. KCs can be targeted by many drug carrier systems (including nanoparticles) due to their characteristic scavenging capacity (168). This concept was validated in studies targeting macrophages and KCs with anti-inflammatory dexamethasone and thus reducing hepatic fibrosis in rodent models (169, 170). Furthermore, cholesterol lowering drugs such as ezetimibe or atorvastatin were shown to reduce the accumulation of cholesterol in murine KCs resulting in the resolution of NASH (171). In a more specific approach, inhibiting the lectin galectin-3 has yielded promising results in animal models and is currently being evaluated in clinical trials (172).

## Conclusions

Accumulating research from recent years has revealed an immense heterogeneity of myeloid subsets with diverse roles in homeostasis and disease. Myeloid-derived cells hold key functions in metabolic diseases, particularly in the establishment and progression of fatty liver disease. Activation, differentiation as well as polarization of immune cells are significantly influenced by distinct local and systemic signals, and the progression of NAFLD is driven by inflammatory immune cells. Importantly, there is a orchestrated interplay between myeloid cells in different compartments, such as circulation/bone marrow, gut, adipose tissue, and liver. In particular, the functional and phenotypic versatility of myeloid cells plays a central role in the pathology of NAFLD and related diseases. Targeting myeloid cells to overcome metabolic disorders has therefore emerged as a promising approach in treating fatty liver disease.

## Author contributions

JH wrote the manuscript and designed the figures. OK contributed to the manuscript text, reviewed, and edited the article. FT reviewed, edited, and finalized the review. All authors approved the manuscript and agreed to be accountable for the content of the work.

### Conflict of interest statement

FT's work in the laboratory has received fundings by Tobira/Allergan, Galapagos, Inventiva and Bristol Myers Squibb.

The remaining authors declare that the research was conducted in the absence of any commercial or financial relationships that could be construed as a potential conflict of interest.

## Abbreviations

DAMP: danger-associated-molecular pattern
DCs: dendritic cells
FXR: farnesoid X receptor
GPR: G-protein-coupled-receptor
IL: interleukin
JNK: c-jun N-terminal kinase
KC: Kupffer cell
LPS: lipopolysaccharide
LXR: liver X receptor
MAPK: mitogen-activated protein kinase
NAFLD: non-alcoholic fatty liver disease
NASH: non-alcoholic steatohepatitis
NF-κB: nuclear factor ‘kappa-light-chain-enhancer’ of activated B-cells
NLRP3: NOD-like receptor protein 3
NOD: nucleotide oligomerization domain
PAMP: pathogen-associated-molecular pattern
PPAR: peroxisome proliferator-activated receptor
PRR: pattern recognition receptor
PUFA: polyunsaturated fatty acids
ROS: reactive oxygen species
SCFA: short chain fatty acids
STAT: signal transducer and activator of transcription
TLR: toll-like receptor
TNF: tumor necrosis factor
TRAIL: TNF related apoptosis inducing ligand.
